# Synergistic anti-tumor therapy by a comb-like multifunctional antibody nanoarray with exceptionally potent activity

**DOI:** 10.1038/srep15712

**Published:** 2015-10-28

**Authors:** Huafei Li, Yun Sun, Di Chen, He Zhao, Mengxin Zhao, Xiandi Zhu, Changhong Ke, Ge Zhang, Cheng Jiang, Li Zhang, Fulei Zhang, Huafeng Wei, Wei Li

**Affiliations:** 1 International Joint Cancer Institute, the Second Military Medical University, Shanghai 200433, China; 2 State Key Laboratory of Antibody Medicine and Shanghai Key Laboratory of Cell Engineering, Shanghai 200433, China; 3 PLA General Hospital Cancer Center, PLA Graduate School of Medicine, Beijing 100853, China

## Abstract

Simultaneously blocking multiple mediators offers new hope for the treatment of complex diseases. However, the curative potential of current combination therapy by chronological administration of separate monoclonal antibodies (mAbs) or multi-specific mAbs is still moderate due to inconvenient manipulation, low cooperative effectors, poor pharmacokinetics and insufficient tumor accumulation. Here, we describe a facile strategy that arms distinct mAbs with cooperative effectors onto a long chain to form a multicomponent comb-like nano mAb. Unlike dissociative parental mAbs, the multifunctional mAb nanoarray (PL-RB) constructed from type I/II anti-CD20 mAbs shows good pharmacokinetics. This PL-RB simultaneously targets distinct epitopes on a single antigen (Ag) and neighboring Ags on different lymphocytes. This unique intra- and intercellular Ag cross-linking endows the multifunctional mAb nanoarray with potent apoptosis activity. The exceptional apoptosis, complement-dependent cytotoxicity (CDC), antibody-dependent cellular cytotoxicity (ADCC) that are synchronously evoked by the nano PL-RB are further synergistically promoted via enhanced permeability and retention (EPR), which resulted in high intratumor accumulation and excellent anti-lymphoma efficiency.

By targeting tumor-specific and/or -associated antigens (TSAs/TAAs), monoclonal antibodies havehave revolutionized the treatment of many human malignancies with considerable clinic success^1^,^2^. Since anti-CD20 mAb (rituximab) was approved by Food and Drug Administration (FDA) in 1997, more than ten mAbs have been used for cancer therapy^2^,^3^. For further boosting mAbs’ cellular cytotoxicity, many strategies have been developed by conjugation with radionuclides^4^,^5^, chemotherapeutic drugs^6^,^7^, toxins^8^,^9^, enzymes^10^,^11^, and immune factors^12^,^13^,^14^,^15^. All of these modalities are designed and engineered to increase the binding avidity with enhanced tumor toxicity.

It is known that multiple mediators participate in the overall pathogenesis of complex diseases via distinct or redundant mechanisms^16^,^17^,^18^. Simultaneously blocking two or more effector molecules may yield a better therapeutic index than the inhibition of single target^16^,^17^. Thus, multivalent antibodies by DVD and crossMAb technology^19^,^20^,^21^ and the combined therapy by two or more mAbs have been developed for targeting distinct antigens and/or signals^22^,^23^. The combination of trastuzumab with pertuzumab has been approved for clinical use for blocking distinct human epidermal growth factor receptor (HER) signaling pathways. Their curative potential is still moderate, partly because of the complex *in vivo* mechanism^24^,^25^. Many tumors, particularly solid tumors, are inaccessible or insensitive to the deliverable doses of mAbs, with typically long blood circulation times and large mAb dosages^25^,^26^. This inevitably leads to high-level accumulation in normal organs and the quick exhaustion of complements, resulting in undesirable side effects and low ADCC and CDC^27^,^28^,^29^. Importantly, the *in vivo* cooperative effects of two or more drugs can be further enhanced if the separate chronological administration is replaced by simultaneous administration with en masse tumor accumulation^30^.

The development of nanotechnology and nanomaterials offers new hope for meeting the above-mentioned demands^31^ with the potential advantages including facile construction; reducing the mAb dosage; significantly promoting intratumor accumulation and cellular antigen interactions with controllable pharmacokinetics and low side effects^30^,^32^,^33^,^34^,^35^. Most importantly, grouping different types of mAbs onto a single long chain to form a comb-like nano mAb array, that is, the nano mAbs can simultaneously target distinct epitopes on a single antigen or on neighboring antigens, or touch different epitopes on different cells. This unique intra-/intercellular antigen cross-linking may evoke some unexpected cytotoxicity, such as apoptosis. The dilemma that has appeared in the traditional multiple target blocking and combination therapy can thus be successfully surmounted by the nano mAb array, with obviously enhanced *in vivo* synergistic effects, potent activity and low cost.

The clinical application of such mAb nanoarray can be further synergistically promoted with improved pharmacokinetics and an enhanced permeability and retention effect (EPR) by finely tailoring its structure and physicochemical properties^36^,^37^. Therefore, the nano-based mAbs with synergistic effects may represent a new generation of multifunctional mAbs. In this study, the classical type I and type II anti-CD20 antibodies, which target distinct epitopes of the CD20 molecule, are sampled and facilely armed to a long polymer chain to form a novel, multifunctional comb-like anti-CD20 mAb nanoarray (PL-RB). The simultaneous PL-RB-induced intra- and intercellular linking can activate CDC, ADCC and apoptosis. The *in vivo* synergic antitumor activity of PL-RB is systemically investigated in disseminated and localized xeno-transplant human NHL models.

## Results

### Fine construction and characterization of nano-based antibody

**Figure 1:**
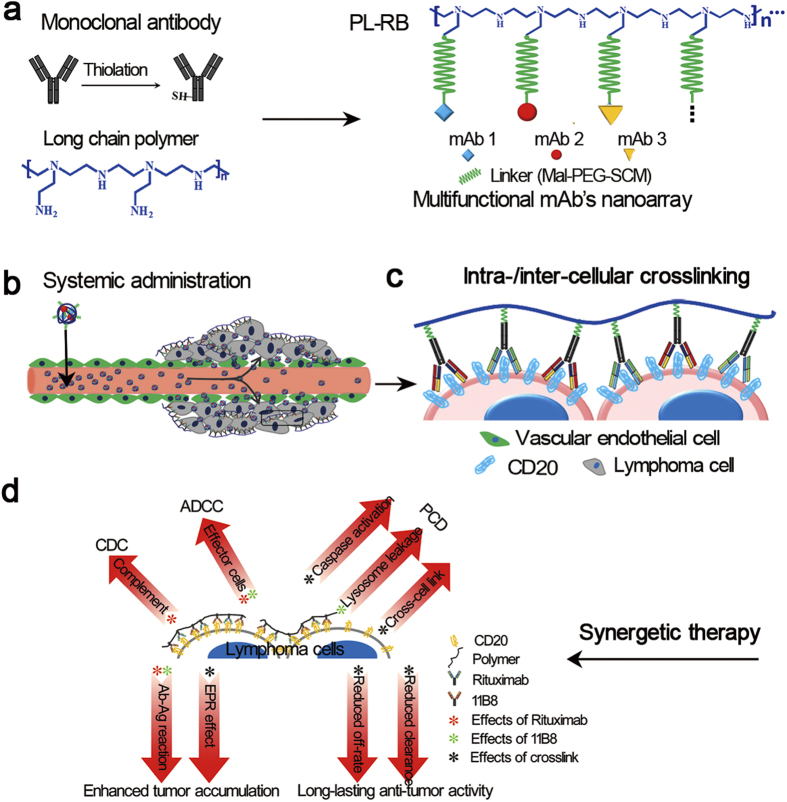
Scheme illustrated the design and fabrication of the comb-like anti-CD20 mAbs nanoarrays (PL-RB). (**a**) The type I and II Rituximab and 11B8 were grafted to the single long polymer chain PEI. (**b**) The EPR effect of nano seized PL-RB. (**c**) The cellular interaction between PL-RB and antigens resulted in intra-/inter- cellular corsslinking. (**d**) The synergetic therapeutic mechanism of CDC, ADCC, PCD induced by PL-RB.

**Figure 2:**
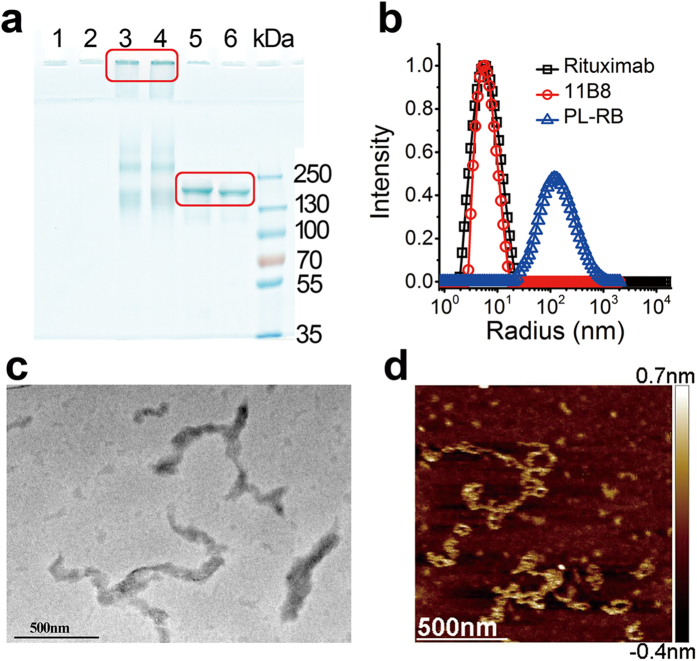
Characterization of PL-RB. (**a**) Parental mAbs successfully anchored to PEI polymer was confirmed by SDS-PAGE under non-reducing conditions. Lane 1 and 2, PEI, lane 3 and 4, PL-RB, lane 5, Rituximab, lane 6, 11B8. (**b**) Size and size distribution of Rituximab, 11B8 and PL-RB. (**c**) Morphology of PL-RB by TEM. (**d**) Morphology under an Atomic force microscopy. Scale bar: 500 nm (right panel).

The schematic for the construction of a comb-like antibody nanoarray (PL-RB) was shown in Fig. 1a. Two or more types of different antibodies with cooperative effects can be grafted together by this facile strategy. Here, the type I and type II anti-CD20 antibodies (rituximab and 11B8) were sampled because they can target distinct epitopes on a single antigen or on neighboring antigens, or epitopes on different cells. The rituximab and 11B8 antibodies were armed to a single long poly(ethylene amine) (PEI) chain as a result of a multifunctional comb-like anti-CD20 nanoarray (PL-RB) as shown in Fig. 1a. The details of the chemical modification and chain grafting are shown in Fig. S1. The successful introduction of antibodies to the long PEI chain was tested by XPS as shown in Fig. S2. Introduction both mAbs and the PEG segment (from the linker) obviously lowered the cytotoxicity of PEI. The high molecular weight (Mw) PL-RB nano-array was confirmed by SDS-PAGE (Fig. 2a). Comparing the polymer (lane 1 and 2) with free rituximab and 11B8 (lane 5 and 6), the obviously retarded bands for PL-RB in lane 3 and 4 clearly showed that the PL-RB was successfully constructed. As illustrated in S1, the average number of mAbs along one polymer chain is larger than 100 (estimated by the static laser light scattering experiment, data not shown here). This is very important because the larger the nanoarray is, the better the efficiency will be. This nanoarray with large M_W_ was further tested by dynamic laser light scattering (LLS). The size of rituximab and 11B8 was smaller than 10 nm, but the size of the PL-RB was approximately 170 nm (Fig. 2b). The comb-like morphology of PL-RB was also confirmed by TEM (Fig. 2c) and AFM (Fig. 2d).

### Unique epitope cross-linking ability of PL-RB

**Figure 3:**
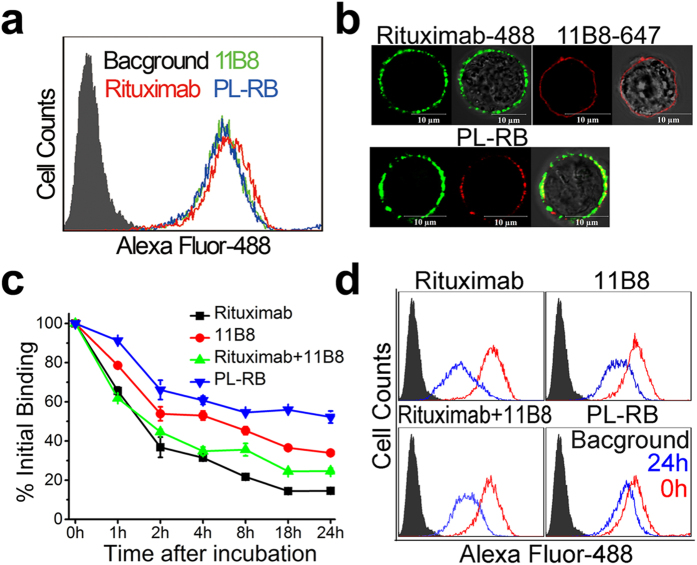
Biorecognition of PL-RB on surface CD20 of Raji cells. (**a)**,(**b**) Binding avidity of PL-RB and parental mAbs to surface CD20 of Raji cells. (**a**) Raji cells were incubated with 10 μg/ml Rituximab, 11B8 and PL-RB and labeled with GAH-488. The cell-bound avidity was evaluated by the MFI of FL-1 and detected by FCM. Black histogram: PBS; Red histogram: Rituximab; Green histogram: 11B8; blue histogram: PL-RB. (**b**) Raji cells were incubated with 10 μg/ml Alexa Fluor 488/647 labeled Rituximab/11B8 and PL-RB on ice for 1 hour and then assessed by confocal microscope. (**c**) Dissociation of PL-RB and parental mAbs from Raji cells. Cells were incubated with 10 μg/ml Rituximab, 11B8 and PL-RB, washed and resuspended in culture medium. Samples were taken at 0, 1, 2, 4, 8, 18 and 24 hours, washed, labeled with GAH-488 and analyzed by FCM. Left panel: Numerical data representing the remaining percent of initial binding mAbs or PL-RBs after different time intervals. Data are mean ± SD of at least 3 experiments. Right panel: The histogram represents the fluorescence intensity distribution of Raji cells. Black histogram shows PBS treated cells. (**d**) Red and blue histograms show the fluorescence intensity distribution after 0 and 24 hours, respectively.

Rituximab and 11B8 were labeled by Alexa Fluor-488 and Alexa Fluor-647, respectively. The FCM results (Fig. 3a) showed that the binding activity with Raji cells of PL-RB was stronger than that of the parental mAbs, which was further evaluated by CLSM (Fig. 3b). The green red fluorescence on the cellular surface was from the rituximab and 11B8. The above results showed that the binding ability of the nanoarray was higher than that of the dissociated mAbs.

The “off-rate” experiment was demonstrated by comparing the dissociation of PL-RB and its parental mAbs from Raji cells. As shown in Fig. 3c,d, approximately 52.23 ± 3.09% of the PL-RB remained on Raji cells after 24 hours. While the remaining ratio of rituximab, 11B8 and the rituximab + 11B8 blend was approximately 14.54 ± 0.12% (**p = 0.001), 33.90 ± 1.39% (**p = 0.005) and 24.64 ± 1.48% (**p = 0.005), respectively. It is worth noting that the remaining ratio of 11B8 was more than that of rituximab (**p = 0.001). The “off-rate” of PL-RB was much lower than the parental mAbs clearly showed high epitope cross-linking ability of nano based antibody^34^.

### *In vitro* potent anti-lymphoma activity

**Figure 4:**
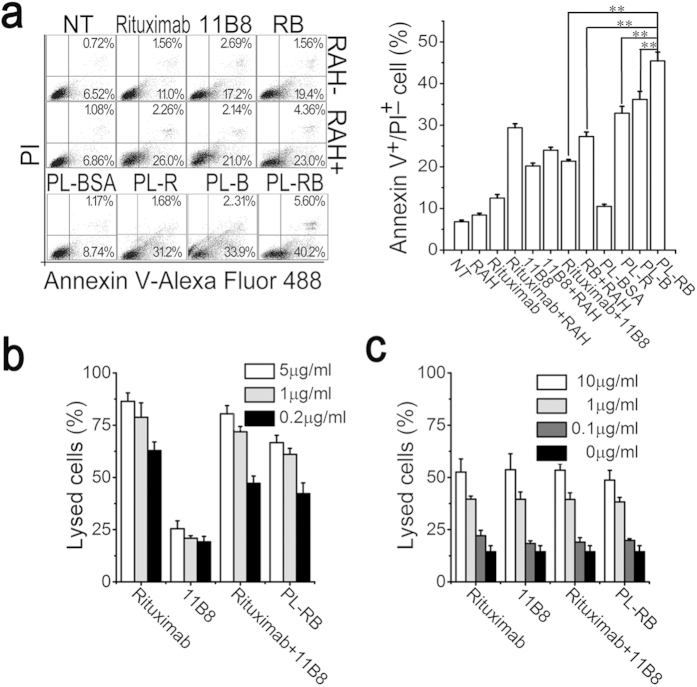
*In vitro* anti-tumor activity of PL-RB. (**a**) Apoptosis-inducing ability of anti-CD20 mAbs and PL-RB. Apoptotic cells were assessed by FCM following staining with Alexa Fluor 488 anti-Annexin V & PI. Apoptotic plot was shown in the left panel and quantitative analysis of apoptotic cells among groups was shown in the right panel (**p < 0.01). Data are expressed as means ± SD (n = 3). (**b**) CDC activity against Raji cells. Cells were incubated with 0.2, 1, 5 μg/ml anti-CD20 mAbs and PL-RBs, supplemented with 5% (v/v) fresh human serum. Data are expressed as means ± SD (n = 3). (**c**) ADCC activity against Raji cells. Cells were incubated with 0.1, 1, 10 μg/ml anti-CD20 mAbs and PL-RBs, supplemented with human PBMCs as effector cells at an E:T ratio of 25:1. Data are expressed as means ± SD (n = 3).

The corresponding cooperative anti-tumor activity of PL-RB was evaluated via CDC, ADCC and programmed cell death (PCD). The *in vitro* apoptosis inducing ability were evaluated and compared among the NT, RAH, R, B, R (RAH), B (RAH), RB (blend), RB (linked by RAH), PL-R, PL-B, PL-BSA, PL-RB as shown in Fig. 4a. The rituximab- and 11B8-induced PCD were 12.51 ± 0.86% and 20.20 ± 0.71%, respectively. When cross-linked by the RAH, the PCD-inducing ability in Raji cells by the two mAbs was enhanced by a small amount (**p < 0.01). Moreover, the PCD-promoting efficiency of rituximab was much higher than 11B8. Interestingly, the PL-RB-induced PCD was increased approximately 5-fold, which was remarkably higher than all other tested samples. Notably, the PCD by PL-RB was approximately 30% higher than that of the non-cross-linked RB or RB-RAH at the same dosage, which indicated that linking both R and B together for the anti-NHL therapy is necessary. As mentioned above, rituximab (type I) held potent ability to mediate CDC, but the PL-RB appeared to be less effective in mediating CDC as compared to the parental mAbs (Fig. 4b). In contrast, the ability of PL-RB to mediate ADCC was not affected compared to the parental mAbs as shown in Fig. 4c.

**Figure 5:**
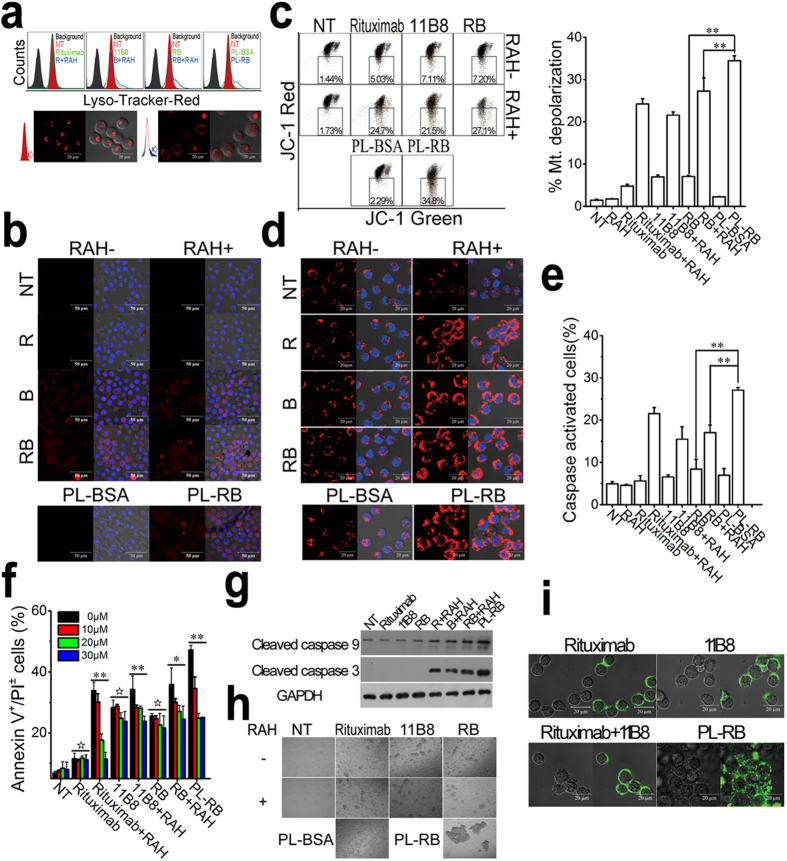
*In vitro* potent anti-lymphoma activity by PL-RB. (**a**-**b**) Lysosome related: (**a**) The volume of the lysosomal compartment was measured by FCM and CLSM after labeling with LysoTracker-red probe. (**b**) Confocal microscopy of cathepsin B staining (red). DNA was counterstained with DAPI (blue). (**c**-**d**) Caspase related: (**c**) Detection of mitochondrial membrane potential (MMP) indicated by decrease of fluorescence intensity of FL-2 (JC-1 Red) (gate of the left panel). The percentage of cells with mitochondrial depolarization was shown in the right panel. Data are expressed as means ± SD (n = 3). (**d**) Effect of anti-CD20 mAbs and PL-RBs on Cyto chrome c release by CLSM. Cells were fixed and labeled for Cytochrome c (red) and DNA (blue). (**e**) Caspase activation in PL-RB induced apoptosis indicated by FLICA reagent in FCM. The percentage of caspase activativation was calculated as mean ± SD (n = 3). (**f**) Apoptosis can be partly inhibited by a caspase inhibitor (Z-VAD-FMK). Raji cells were treated for 30 minutes with 0, 10, 20, 30 μM Z-VAD-FMK before addition of mAbs and PL-RBs. Data are expressed as mean ± SD (n = 3) (**p < 0.01, *p < 0.05, ^☆^p > 0.05). (**g**) Western Blotting analysis of cleaved caspase 3/9 of Raji cells treated with PL-RB and parental mAbs. (**h**-**i**) Effect of PL-RB and parental mAbs on the crosslinking of Raji cells indicated by light microscopy (**h**) or confocal microscope following staining with Alexa Fluor-488 goat anti-human secondary antibodies (**I**).

The mechanisms related to the excellent PCD inducing ability of PL-RB predicted above were examined for the caspase-dependent and -independent pathways. For testing the caspase-independent pathway (Fig. 5a), a lysosome tracker was used to label the lysosomes in PL-RB-treated cells. The FCM results revealed that the distribution of FL-2 (LysoTracker) in Raji cells exhibited a visible red shift after 16-hour incubation with 11B8 and its cross-linked groups by RAH. The largest red shift appeared in the PL-RB group. Furthermore, the CLSM images clearly showed that there was discrepancy of LysoTracker signal in PL-RB group (bottom panel, Fig. 5a). The relatively small lysosomes in normal cells (left side) were swollen resulting in enlarged compartments as labeled by diffusion of the red fluorescence to the cytoplasm (right side). This was similar to the lysosomal-mediated PCD reported in previous publications^34^,^35^. The IF staining for cathepsin B was further conducted to confirm the lysosome compartment collapse, which is known as a lysosomal component in Fig. 5b. Confocal microscopy revealed that a substantial increase of cathepsin B (red fluorescence) throughout the cytoplasm of PL-RB-treated cells, which was in accord with the results of lysosome tracker labeling.

The mitochondrial depolarization, which was related to the caspase activation, was evaluated by FCM following JC-1 staining in PL-RB-treated cells^38^,^39^. Cells with mitochondrial depolarization were characterized by the decrease of FL-2 (JC-1 red) fluorescence intensity. As shown in Fig. 5c, the mitochondrial depolarization in the RAH cross-linked mAb-treated group was higher than that of rituximab (**p = 0.001) and 11B8 (**p = 0.007). At the same conditions, the mitochondrial depolarization in the PL-RB group was remarkably higher than the free (**p = 0.003) and RAH cross-linked (rituximab + 11B8) (**p = 0.001) groups. Subsequently, Cyt-c release, which also indicated the depolarization, was determined by IF labeling as shown in Fig. 5d. The dotted pattern of the NT group indicated the Cyt-c location in the mitochondria^40^. The diffuse pattern with Cyt-c translocation into the cytoplasm clearly showed mitochondrial changes in PL-RB-treated or RAH cross-linked mAb-treated cells, which was similar to the JC-1 staining.

Caspase activation was confirmed by FCM as shown in Fig. 5e. Similarly, the caspase was not significantly activated by the free anti-CD20 mAbs. Approximately 20% caspase activation was observed when the rituximab or 11B8 was cross-linked by RAH. However, compared to the free (**p = 0.001) or RAH cross-linked (**p = 0.007) groups, a remarkable increase of caspase activation appeared in the PL-RB-treated cells (approximately 28%). In contrast, the apoptosis inhibition results (Fig. 5f) revealed that 10 to 30 μM ZVAD-FMK (a caspase inhibitor) can prevent the apoptosis induced by RAH cross-linked anti-CD20 mAbs in a dose-dependent manner (**p < 0.01, *p < 0.05, ^☆^p > 0.05). Additionally, compared to the other groups, the PL-RB-evoked PCD can be significantly reduced by ZVAD-FMK (**p = 0.005). Such caspase activation results were further verified by western blotting analysis (Fig. 5g). All of the above results clearly showed that PL-RB can induce PCD via both caspase-dependent and -independent pathways.

An interesting phenomenon was found in the experiments, that is, large intercellular clusters appeared in PL-RB-treated Raji cells. To confirm and identify the nature of this interesting phenomenon, Raji cells were incubated with anti-CD20 mAbs and PL-RBs at same mAb concentration −2.5 μg/mL. Figure 5h showed that few inter-cellular clusters were observed in the rituximab-treated group. And a few clusters appeared in the 11B8-treated group. Crosslinking with RAH made no significant difference. In contrast, the large inter-cellular clusters appeared in the PL-RB-treated Raji cells. These interesting PL-RB-induced intercellular clusters, which were similar to HA^41^,^42^, were further clearly visualized by confocal microscopy (Fig. 5i). This intercellular cross-linking ability was also found as we shortened the chain length (another nanoarray PS-RB) (**S3**). We thought that these interesting inter-cellular clusters could also contribute to the potent PCD, which was discussed in the following section.

### *In vivo* pharmacokinetics analysis of the PL-RB nanoarrays

**Figure 6:**
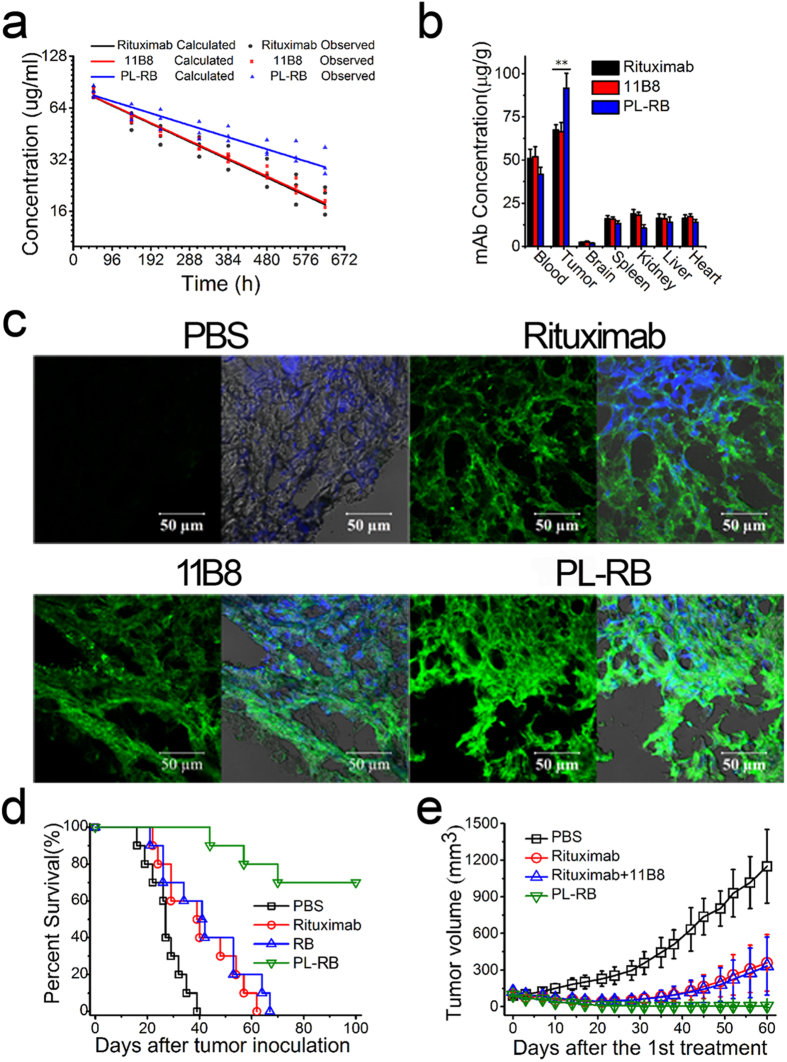
*In vivo* pharmacokinetics analysis and therapeutic efficiency of PL-RB. (**a**-**b**) *In vivo* distribution of PL-RB in lymphoma bearing SCID mice. Tumor bearing mice were injected via tail vein with Rituximab, 11B8 or PL-RB for. After 24 hours, mice were sacrificed and different tissue samples were collected and analysis. (**a**) Time dependence of Rituximab, 11B8 and PL-RB concentration. Line was the calculated value and dot shows the observed value from 3 mice/group. (**b**) Therapeutic mAb concentration in different tissue samples were determined by ELISA. Data are the mean ± SEM derived from separate organs of three different animals. (**c**) Frozen section from tumors were stained by DAPI (blue) and Alexa Fluor 488 goat anti-human secondary antibodies (green) as visualized by CLSM. (**d**) The survival of tumor-bearing SCID mice. SCID mice were injected with 1 × 10^7^ Raji cells via tail vein on day 0, followed by the treatment of Rituximab, Rituximab +11B8 and PL-RB on day 3, 5, 7, 9 and 11. The survival curves were plotted according to the Kaplan-Meier method and compared using the log-rank test. (**e**) Groups of SCID were injected subcutaneously with 2 × 10^7^ Daudi cells and treated with Rituximab, Rituximab + 11B8 and PL-RB. Tumor size was measured 2-dimensionally with a caliper and tumor volume was shown as mean ± SD (n = 3).

For the pharmacokinetics (PK) assays, a one-compartment model was used to describe the time course of the blood concentrations. These two humanized antibodies are developed for treating human diseases, it is necessary to investigate the characteristics of the modulate with human IgG constant regions in the mouse or rat models, which is similar to many previous studies reported by other groups^19^. As shown in the concentration-time curve (Fig. 6a) and PK parameters (Table S1), the elimination half-time (t_1/2_) of PL-RB was 429.0 ± 37.6 hours, which was much longer than the 274.8 ± 30.1 hours for rituximab (**p = 0.005) and 278.4 ± 9.0 hours for 11B8 (**p = 0.001). The prolonged t_1/2_ of approximately 10 days for PL-RB directly demonstrated that the elimination of PL-RB from mouse peripheral blood was much slower than the free mAbs.

For the *in vivo* distribution determination, PL-RB and the corresponding parental mAbs were administered via tail vein to the mice bearing lymphoma. The mice were euthanized 24 hours post-administration. The major organs, including tumor, brain, blood, heart, kidneys, liver and spleen, were partially harvested, weighed, and assayed to evaluate the mAb concentrations in μg mAb/g tissue. Figure 6b revealed significant increases in tumor accumulation of PL-RB compared with rituximab (**p = 0.005) and 11B8 (**p = 0.004). Additionally, the frozen sections of the tumor tissues were prepared for IF staining and observed by CLSM (Fig. 6c). The tumor accumulation of PL-RB was significantly increased as indicated by the increase of green color, which was consistent with the ELISA results.

### Therapeutic efficiency of PL-RB in disseminated and localized xeno transplant tumor model

Both the disseminated and localized human NHL xeno transplant models were prepared to evaluate the *in vivo* lymphoma depletion efficacy of PL-RB. In the disseminated model, lymphoma-bearing mice were randomly administered tail vein injections of PBS, rituximab, rituximab +11B8 and PL-RB every other day for 5 treatments. The survival curves are shown in Fig. 6d and the statistical analysis results in Table S2. The rituximab-treated group had a longer survival time than the control group that was injected with PBS (*p = 0.010). The combination therapy of rituximab plus 11B8 showed similar results (**p = 0.007), which were not significantly different from the rituximab group (p = 0.494). Surprisingly, compared to the RB and rituximab groups, PL-RB significantly prolonged the survival time, with a CR percentage of 7/10 indicated by long-term survival (>100 days post treatment).

The excellent anti-tumor activity of PL-RB was also confirmed by a localized human NHL xeno-transplant model (Fig. 6e). The rituximab- and/or 11B8-treated groups had similar lymphoma tumor volumes. The tumor volume of the PL-RB-treated group was remarkably lower than the rituximab ± 11B8 group, which was characterized by 3/4 of CR mice with no measurable mass. Noted here, this study is expected to provide a proof of concept that new nano-based antibody generated by facile nanotechnology can significantly enhanced antitumor efficacy. If this modulation is possible to be translated into clinic in the future, the detail immunogenic effects will be clearly investigated. Moreover, it is also interesting to investigate whether such novel antibody could suppress the antibody resistance.

## Discussion

In this work, we described a general and facile approach to develop a multi-component nano-based antibody that can target two or more distinct epitopes simultaneously (Fig. 1a and Table S1). The unique intra-/intercellular antigens cross-linking ability by the comb-like nano antibody can evoke synergistic effects on the tumor cells. Moreover, unlike the traditional combination therapy by two or more separate mAbs^20^,^43^,^44^, the *in vivo* therapeutic efficiency of this mAb nanoarray was obviously and synergistically enhanced by the EPR effect, which can avoid the inconvenient manipulation, good pharmacokinetics and obviously lower the dosage of mAbs as a result of enhanced tumor accumulation (Fig. 1b). Noted here, the therapeutic index of type I anti-CD20 mAb rituximab is still moderate, partly because of its unclear mechanism, low cytotoxicity and poor *in vivo* performance. The mechanisms involved in anti-CD20 antibodies’ therapeutic efficacy are mainly complement-dependent cytotoxicity (CDC), antibody-dependent cellular cytotoxicity (ADCC), and the induction of apoptosis^45^,^46^. The type I mAbs (rituximab) can efficiently promote complement-dependent cytotoxicity (CDC) with relatively low apoptosis. Type II mAbs (tositumomab-like) can efficiently induce programmed cell death (PCD), but are relatively inactive in complement activation. Both type I and II mAbs had similar contributions to antibody-mediated cellular cytotoxicity (ADCC) via FcR-bearing myeloid effectors. Therefore, rituximab and 11B8 are ideal candidates for constructing a multifunctional comb-like mAb nanoarray (PL-RB) by mass arming onto a long PEI chain, which can block distinct epitopes on a single CD20 molecule and/or epitopes of different CD20 molecules on the same or different cells, resulting in intra-/intercellular antigen cross-linking (Fig. 1c). To our knowledge, this is the first study to report a strategy for the construction of a multifunctional mAb nanoarray, that is the nano-based antibody, composed of two or more different antibodies with cooperative effectors.

*In vitro* studies showed that the “off-rate” of PL-RB was lower than its parental mAbs, which was mainly attributed to the large amount of 11B8s mass-arrayed on the cellular surface and, consequently, the enhanced the affinity of rituximab to the NHL cells^47^. Most importantly, compared to the dissociated parental mAbs, PL-RB obviously induced Raji cell adhesion, which resulted in obvious intra-/intercellular antigen cross-linking, as predicted above (Fig. 5i). Here, there may be two types of antigens cross-linked at the intracellular level: single CD20 epitopes and epitopes on different CD20 molecules. The size of the nanoarray was approximately 100 nm, which was much larger than the size of the single antigen (approximately 10 nm). Thus, the cross-linking of the epitopes on neighboring CD20 molecules dominated because it was difficult for two mAbs to spontaneously bind epitopes on a single CD20. At the intercellular level, the neighboring cells were densely collapsed by the long PL-RB, which we termed as “intercellular cross-linking.” Both the intracellular and intercellular antigen crosslinking were highly susceptible to apoptosis, as characterized by mitochondrial depolarization, lysosomal membrane permeabilization and phosphorylation or up- or down-regulation of proteins related to the apoptosis signal transduction pathway^44^,^48^. The PL-RB-induced mitochondrial depolarization was due to caspase activation because the intrinsic pathway of caspase activation triggers mitochondrial depolarization, the release of cytochrome c (Cyt-c) and activation of caspase-9 and caspase-3. The evidence shown in Fig. 5 firmly supported the fact that the intra-/intercellular antigen crosslinking by PL-RB obviously contributed to exceptionally high levels of apoptosis, including a lysosome-related pathway (regulated by the type II mAb, 11B8) and a caspase-dependent pathway (generated by the crosslinking of the type I mAb, rituximab). Caspase activation was important for promoting the cytotoxicity of the anti-CD20 mAbs^40^,^48^. Therefore, it is also helpful to observe that the ADCC, CDC and apoptosis were significantly increased in the PL-RB-treated Raji cells (Fig. 1d).

The high synergistic therapeutic potential of PL-RB was further confirmed by subsequent *in vivo* studies shown in Fig. 6d,e. Both disseminated and localized xeno-transplant human NHL tumor inhibition clearly demonstrated that PL-RB was more effective than the parental mAbs. Taken together, the mechanism of synergistic therapy that integrated targeted therapy and nanomedicine can be clarified as follows. First, the size of PL-RB nanoarray was in the range of 100–200 nm, which enhanced its EPR effects with high tumor accumulation^49^ (Fig. 1b). Tthe enhanced tumor accumulation of PL-RB is mainly attributed to two steps: The first step is the well-kwon EPR effect of PL-RB. The second step is the PL-RB penetration from the tumor spheroids to the tumor cells and consequently binding onto the cellular surface. For the antibody case, the driving force for this microdistribution is mainly determined by the cosmetic pressure and the antigen-antibody interaction. Once PL-RB bound to the NHL cell surface, the reduced “off-rate” from 11B8 gave rise to potent, durable and long-lasting anti-tumor activities. All of the well-known tumor-suppressing pathways, ADCC, CDC and potent apoptosis, were synchronously activated by PL-RB^45^,^49^. Moreover, the intra-/intercellular cross-linking further enhanced the lymphoma cells’ susceptibility to apoptosis (Fig. 1c), where both the caspase-dependent and -independent apoptotic pathways were significantly evoked. In conclusion, construction a novel nano-based antibody by the feasible mass grafting of distinct mAbs to one long chain for blocking multiple-disease mediators, which resulted in exceptional anti-tumor effectors and was synergistically promoted by the EPR effect and cellular-level antigen cross-linking (Fig. 1d). Thus, the curative efficiency of the combination therapy was significantly improved by the convenient manipulation, clear *in vivo* mechanism, good pharmacokinetics and sufficient tumor accumulation and synergistic effectors, which indicated that this facile and fashionable strategy would be a highly potent cancer therapy.

## Methods

### Cell lines, materials and animals

Two human B lymphoma cell lines, Raji and Daudi, were obtained from the American Type Culture Collection (ATCC, Manassas, VA 20110 USA). The cells were propagated and maintained in RPMI 1640 medium supplemented with 10% (v/v) heat-inactivated fetal bovine serum (FBS, GIBCO, USA). Rituximab was purchased from Roche, and 11B8 was expressed and purified in our laboratory as previously described^14^,^26^. The rabbit anti-human IgG F(ab’)_2_ fragments and polyethylenimine (PEI, 50 wt.%) were purchased from Sigma-Aldrich (USA). The maleimide-PEG-SCM (MPEGS) was purchased from Greative PEGWorks (USA). Four-week-old healthy female SCID mice and BALB/c nude mice were purchased from the Shanghai Experimental Animal Center of the Chinese Academy of Sciences (Shanghai China) and were housed in specific pathogen-free conditions. Fresh human serum and human peripheral blood mononuclear cells (PBMCs) were voluntarily donated by Prof. Wei Li and approved by the Institutional Review Board of the Second Military Medical University. All of the experiments were performed in accordance with ethical guidelines under the protocols approved by the Committee on Animals of the Second Military Medical University (Shanghai China).

### Preparation of the comb-like anti-CD20 mAb nanoarray (PL-RB)

First, rituximab, 11B8 and BSA (used as control) were thiolated by dithiothreitol, resulting in rituximab ~SH, 11B8 ~ SH and BSA~SH, as described in our previous publications^33^. Then, the PEI polymer (M_W_ approximately 75 kDa) was dissolved in phosphate buffered saline (PBS) at a concentration of approximately 1 mg/mL. The MPEGS linking agent (N_PEI_/N_MPEGS_ approximately 1:20) was added to the polymer solutions with stirring and N_2_ bubbling for approximately 4 hours at room temperature (RT). The unreacted MPEGS was removed by dialysis. Then, an equal amount of thiolated rituximab and 11B8 were slowly dropped into a 5-ml test tube containing the MPEGS-PEI solution (mAb/PEI mass ratio = 1000:3.44). The reaction was conducted under N_2_ with stirring for 6–8 hours. The un-grafted mAbs were separated by dialysis. The comb-like anti-CD20 nanoarrays (PL-RB) obtained from this procedure are shown in Fig. 1. The control sample, PEI-MPEGS-BSA (PL-BSA), was constructed in the same way. Purified PL-RB was quantified by NanoVue^TM^ (GE Healthcare) and analyzed on an 8% SDS-PAGE gel followed by Coomassie brilliant blue staining.

### Size and morphology characterization

The size and size distribution were tested by the dynamic laser light scattering instrument (DLLS, ALV/CGS-3, Germany) at the scattering angle of 30°^33^,^36^. The PL-RB morphology was characterized by TEM and AFM. To prepare stained specimens for TEM (Hitachi, H-7000 Electron Microscope), a PL-RB solution (0.5 mg/ml) was dropped onto a 200-mesh Formvar-free carbon-coated copper grid (Ted Pella Type-A). After water evaporation, the sample was inversely covered onto a small drop of 2% hydrodated phosphotungstate (PTA) solution. The conventional TEM images were obtained at 100 kV. For the AFM experiments, 10 ml of the PL-RB stock solution was spread onto freshly cleaved mica followed by a gentle rinse. The sample was air-dried and mounted onto the XY scanning station. The images were obtained by an atomic force microscopy (AFM, Autoprobe CP Research, Thermomicroscopes, Veeco Instruments Inc.).

### Confocal laser scattering microscopy (CLSM)

For CLSM, the harvested cells were placed onto poly-D-lysine-coated (Sigma-Aldrich) microscope slides. The samples were fixed with 4% paraformaldehyde and permeabilized with 0.3% Triton X-100. The slides were then stained using the appropriate antibodies and probes. After washing, the samples were observed using a confocal microscope (Zeiss LSM 710, Germany).

### Binding avidity

The binding activity was assessed by flow cytometry (FCM) and CLSM. For the FCM evaluation, 10 μg/mL of rituximab, 11B8 or PL-RB were incubated with Raji cells for 1 hour on ice. Then, the samples were rinsed with PBS and labeled with an Alexa Fluor-488 goat anti-human IgG secondary antibody (GAH-488, 1:500, Invitrogen). After washing, the mean fluorescence intensity (MFI) of FL-1 was obtained using a FACScan flow cytometer (Becton Dickinson, San Jose, CA). CLSM was also used to visualize the binding profile of PL-RB.

### Off-rate measurement

The harvested Raji cells were incubated with 10 μg/mL rituximab, 11B8 or PL-RB for 1 hour. Then, the cells were washed and resuspended in culture medium. After different time intervals, the samples were collected, washed, stained with GAH-488 and analyzed by FCM. The percentage of initial binding was calculated by the following equation:


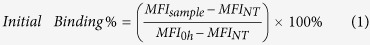


where MFI_sample_, MFI_NT_ and MFI_0h_ are the mean fluorescence intensity of sample, the negative control and the sample at 0 hour, respectively.

### Annexin V/PI staining.

The cells were incubated with 10 μg/mL rituximab, 11B8 or PL-RB for 16 hours. After washing, the cells were stained with Alexa Fluor-488 Annexin-V and propidium iodide (PI) (Invitrogen) and analyzed by two channel-FCM of FL-1 (Annexin-V) and FL-2 (PI). For cross-linking, 20 μg/mL of rabbit anti-human IgG F(ab′)_2_ fragments were added to the samples one hour after the addition of the free mAbs. For the apoptosis inhibition assays, different concentrations of a caspase inhibitor (Z-VAD-FMK, Promega) were added before the addition of the free mAbs or PL-RBs.

### CDC and ADCC

Raji cells were incubated anti-CD20 mAbs or PL-RBs. For the CDC assays, 5% (v/v) fresh human serum was added as a source of complement. For the ADCC assays, human peripheral blood mononuclear cells (PBMCs) were added as effector cells (effector/target = 25:1). After a 4-hour incubation, the lysed cells were recorded by the mean luminescence intensity (MLI) as determined by the CytoTox-Glo™ Cytotoxicity Assay kit (Promega). Cells lysed by the lysis reagent were used as positive controls. The percentage of lysis was calculated according to the following equation:


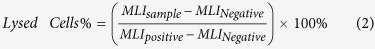


where MLI_sample_, MLI_Negative_ and MLI_positive_ are the mean luminescence intensity of sample, the negative control and positive sample, respectively.

### Lysosomal permeability

Raji cells were treated with 10 μg/mL of the anti-CD20 mAbs or PL-RBs for approximately 16 hours. Then, the cells were labeled with 200 nM LysoTracker Red DND (Invitrogen) at 37 °C for 30 minutes. The FL-2 fluorescence from the labeled cells was analyzed by FCM and CLSM. Unlabeled cells were used as a background control.

### Mitochondrial membrane potentials (MMP) and caspase activation

Raji cells were incubated with anti-CD20 mAbs and PL-RBs for 16 hours. After washing, the JC-1 probe (Beyotime Biotechnology, Shanghai China) was used to measure mitochondrial depolarization. The Vybrant^®^ FAM Poly Caspase Assay Kit (Invitrogen) was employed to estimate the caspase activation in Raji cells using FCM. Both experiments followed the manufacturer’s protocol.

### Western blotting

Raji cells were incubated with anti-CD20 mAbs and PL-RBs for approximately 16 hours. Then, the cells were harvested, washed and lysed in cell lysis buffer for western and IP (Beyotime Biotechnology, Shanghai China). The cell lysates were subjected to SDS-PAGE and immunoblotted with cleaved caspase-3/9 antibodies (Cell Signaling Technology, USA).

### Homotypic Adhesion (HA) measurement

The cells were incubated with 2.5 μg/mL anti-CD20 mAbs or PL-RBs for approximately 8 hours. Then, the cellular morphology was observed by inverse fluorescent light microscopy. For CLSM, the cells were carefully transferred onto poly-D-lysine-coated microscope slides, labeled with a 1:500 dilution of the GAH-488 antibody and scanned by a confocal microscope.

### Pharmacokinetic analysis

Rituximab, 11B8 or PL-RB (20 mg/kg) was injected into the tail vein of BALB/c nude mice on day 0, 1 and 2, respectively. Then, the mice were randomly divided into three groups of 3 mice each. After different time intervals, 40–60 μL of the venous blood was sampled from the angular vein of the eyes. Enzyme-linked immunoassays (ELISA) were employed to investigate the plasma concentration of the therapeutic mAbs. The data were analyzed by PK solver software^50^.

### *In vivo* distribution

Daudi cells (2 × 10^7^) were subcutaneously inoculated into the lateral flank of 8-week old female SCID mice. When the tumors reached approximately 8 mm in length, 20 mg/kg rituximab, 11B8 or PL-RB was intravenously injected through the tail vein daily for 3 days. Twenty-four hours later, the mice were euthanized. The organs were collected and the frozen sections were stained with GAH-488 and visualized by CLSM. Portions of the major tissues were lysed using TissueLyser-24 (Shanghai Jingxin Experimental Technology, Shanghai, China), and after centrifuging, the ELISA was used to investigate the concentration of the mAbs.

### *In vivo* therapy

For the disseminated models, four groups of 10 eight-week-old female SCID mice were injected with 1 × 10^7^ Raji cells via the tail vein. After 72 hours, the mice were randomly assigned to three groups. Then, rituximab, rituximab +11B8 (1:1) or PL-RB were administered via the tail vein with equal mAb concentrations (15 mg/kg) every other day for 5 treatments. The mice were monitored daily until natural death with a time range of 100 days. All of the surviving animals were euthanized on the 100th day.

For the localized models, Daudi cells (2 × 10^7^) were subcutaneously inoculated into the lateral flank of 8-week-old female SCID mice. When the tumors reached approximately 8 mm in length, 20 mg/kg rituximab, rituximab +11B8 (1:1) or PL-RB was administered via the tail vein weekly for 3 weeks. The tumor size was measured with precision calipers for two perpendicular diameters twice a week and calculated using the following equation:


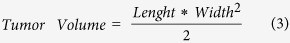


where *Length* and *Width* are the length and width of the tumor as recorded by the calipers.

### Statistical analysis

Statistical analysis was performed by Student’s unpaired t test or ANOVA to identify significant differences unless otherwise indicated. Differences were considered significant at a p value of less than 0.05. ANOVA was used for comparing the difference among three or more groups in the experiments.

## Additional Information

**How to cite this article**: Li, H. *et al.* Synergistic anti-tumor therapy by a comb-like multifunctional antibody nanoarray with exceptionally potent activity. *Sci. Rep.* **5**, 15712; doi: 10.1038/srep15712 (2015).

## Supplementary Material

Supplementary Information
