# Supplementary material for: Synergistic anti-tumor therapy by a comb-like multifunctional antibody nanoarray with exceptionally potent activity
Source: Sci Rep. 2015 Oct 28;5:15712. doi: 10.1038/srep15712 (PMC4623742; doi:10.1038/srep15712)
Supplement: Supplementary Information [file srep15712-s1.pdf]

<Supplementary data>

Synergistic anti-tumor therapy by a comb-like multifunctional antibody nanoarray with exceptionally potent activity

Huafei Li[1], Yun Sun[1], He Zhao[1], Di Chen[1], Mengxin Zhao[1], Xiandi Zhu[1], Changhong Ke[1], Ge Zhang[1], Cheng Jiang[1], Li Zhang[1], Fulei Zhang[1], Huafeng Wei[1] & Wei Li[1,2,3,*]

[1] International Joint Cancer Institute, the Second Military Medical University, Shanghai 200433, China
[2] State Key Laboratory of Antibody Medicine and Shanghai Key Laboratory of Cell Engineering, Shanghai 200433, China
[3] PLA General Hospital Cancer Center, PLA Graduate School of Medicine, Beijing 100853, China

Corresponding author: Wei Li
Tel:+86-21-81870804; Fax: +86-21-81870801
E-mail: liwei@smmu.edu.cn

S1: Details of the chemical suynthesis of PL-RB

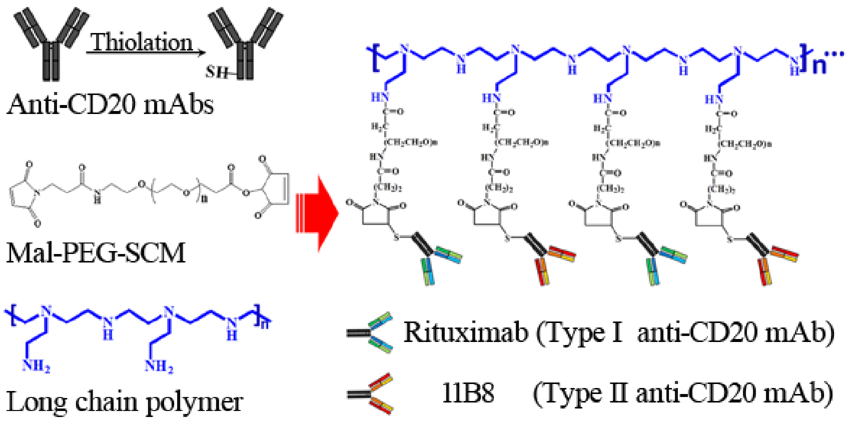

The anti-CD20 mAbs were reduced with 20 mM dithiotheritol for about 1.5 hours under argon at room temperature. The impurity was removed by Sephadex G-150 column (Pharmacia) pre-equilibrated by the deoxygenated buffer 1. Then the solution was dialysis against with the 50 mM HEPES (pH 7.4) resulting in the final reactive targeting moiety anti-CD20-SH which was stored at 4 ^{o}C and N_2 environment for future use. Then linker mal-PEG-SCA was added to the anti-CD20-SH solution at the molar ration 2/1, and stirred at RT and N_2 for about 4 hours. The impurity was removed by dialysis for about 8 hours. The products was anti-CD20-PEG-SCA was then reacted with the PEI at the RT and N_2 for about 2 hours and purified by dialysis against Milli-Q water for 8 hours. Noted here, the molecular weight of PEI is 750000 g/mol, the molecular weight of ethylene amine is 43 g/mol. The number of prime amine N_2 along the polymer chain is thus be calculated as (750000/43)0.3 (~6000). This means that there are about 6000 reactive point for the antibody grafting. The detail number of mAb on the chain was evaluated by the BCA method. It was found that the average number of mAbs along one PEI chain was larger than 100 (data not shown here).

S2: Details of the XPS confirming the mAb's linking

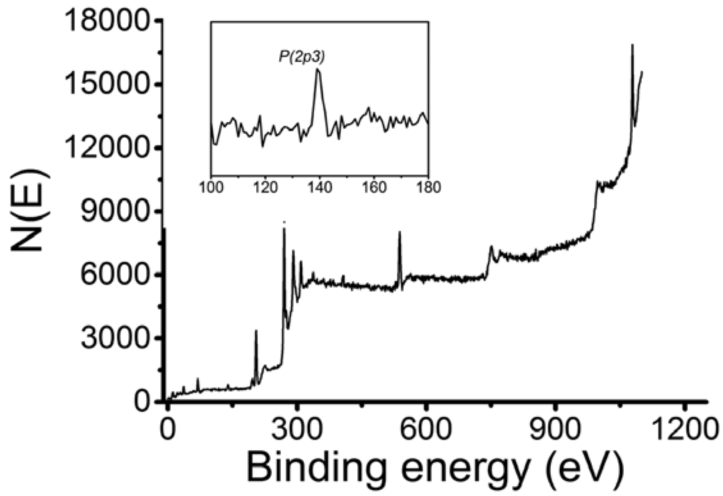

XPS characterization of the PL-RB, which illustrated and confirmated the anti-CD20 mAb's successful grafting onto the PEI chain backbone. (the characteristic XPS peak was shown in the insert Fig.)

S3: Inter-cellular crosslinking by short PS-RB

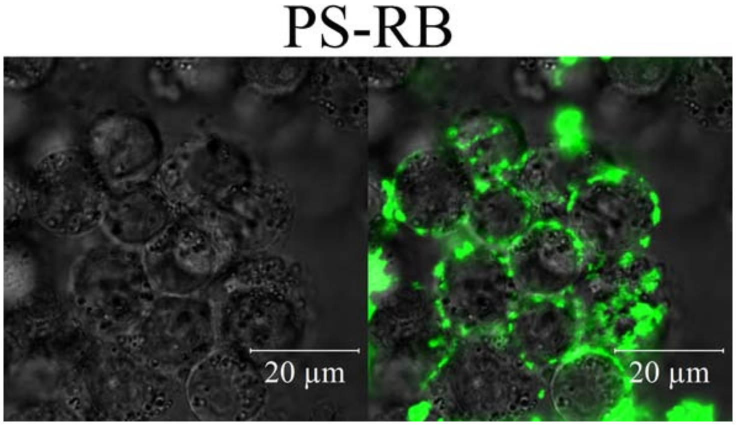

Effect of PS-RB on HA of Raji cells. Cells were treated with 2.5μg/ml PS-RB for 8 hours. Then cell morphology was observed by confocal microscope following staining with Alexa Fluor-488 goat anti-human secondary antibodies.

Table S1: Parameter of pharmacokinetics

Parameter	Rituximab	11B8	PL-RB
**$t_{1/2}$ (h)**	274.8±30.1	278.4±9.0	429.0±37.6
**CL(L/h)**	12.13±2.04	11.83±0.84	7.49±1.01
**MRT (h)**	396.52±43.41	401.58±13.02	618.93±54.33
**Vd (ml)**	4.75±0.27	4.74±0.21	4.60±0.19

$t_{1/2}$： Elimination Half-life

CL:clearance

MRT: mean residence time

Vd： aparent volume of distribution

Table S2: Survival analysis by log rank test

Group	Pair Comparisons of survival						Median survival time (Day)		
	Rituximab		Rituximab+11B8		PL-RB		Mean	SD	95% CI
	Chi-Square	*p* value	Chi-Square	*p*value.	Chi-Square	*p* value			
PBS	6.638	0.010	9.380	0.007	21.837	0.000	26.00	2.64	20.84~31.17
Rituximab	——	——	0.467	0.494	15.613	0.000	39.00	8.70	91.96~56.05
Rituximab+11B8	——	——	——	——	15.056	0.000	41.00	6.33	28.60~53.40
PL-RB	——	——	——	——	——	——	>100	——	>100~

CI: Confidence Interval

S4: The cytotoxicity profile of PEI before and after conjugation with RB

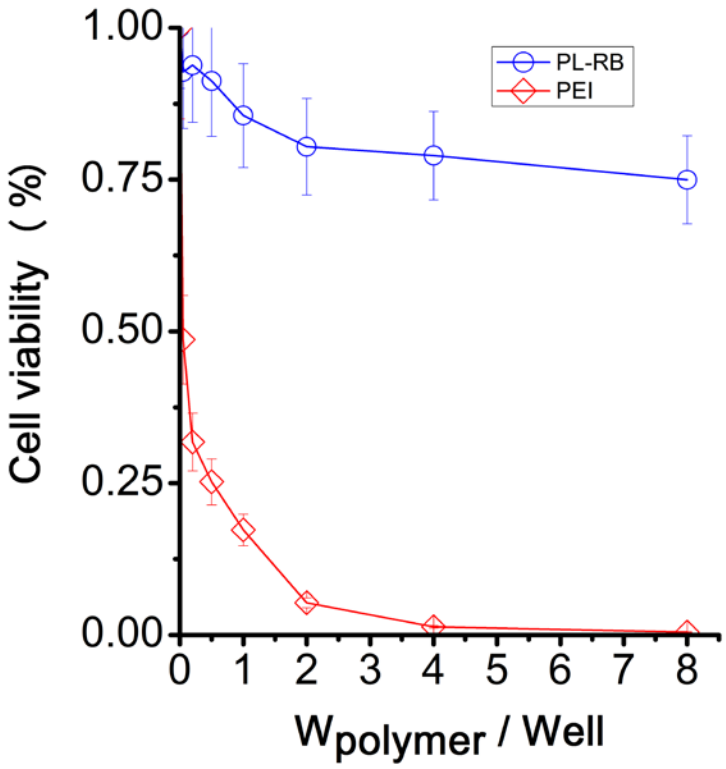

The cytotocity of PEI before and after conjugation with R and B. It can be found that the cytotoxicity of PEI was obviously decreased by conjugation with mAbs. In the well, 5000 293T cells were seeded and coltured for about 12 hours. Then the PL-RB and PEI solutions were added to the 96-well dish with a calculated concentration (0.05, 0.2, 0.5, 1, 2, 4, and 8 ug/well). 24 hours post the culture, the medium was replaced by fresh medium, and the samples were tested by the CC-Kit-8 assay.
